# Bond Analysis of Titanium Rods Embedded in Masonry

**DOI:** 10.3390/ma17071517

**Published:** 2024-03-27

**Authors:** Fitsum Haile, Marco Corradi, Enea Mustafaraj, Harrison Coolledge, Jill Adkins

**Affiliations:** 1 Department of Mechanical and Construction Engineering, Wynne Jones Building, Northumbria University, Newcastle upon Tyne NE1 8ST, UK; f.m.haile@northumbria.ac.uk (F.H.); harrisoncoolledge@gmail.com (H.C.); 2 Department of Engineering and Technology, School of Computing and Engineering, University of Huddersfield, Huddersfield HD1 3DH, UK; 3 College of Engineering and Technology, American University of the Middle East, Egaila 54200, Kuwait; enea.mustafaraj@aum.edu.kw; 4 Research Department, Perryman Company, Houston, PA 15342, USA; jadkins@perrymanco.com

**Keywords:** titanium, earthquake engineering, mechanical testing, masonry

## Abstract

Among the techniques utilized for strengthening masonry structures with advanced materials, the adoption of near-surface mounted (NSM) titanium rods stands out as a promising method for increasing the flexural and shear strength of masonry structures. This method is also known as Bed Joint Reinforcement. Ensuring an effective performance of this technique hinges on establishing a strong bond between the NSM reinforcement and the substrate masonry material. The primary objective of this project was to study the mechanics of this bond using NSM threaded and smooth titanium rods while scrutinizing the impact of key parameters on bond performance. Variables under investigation encompassed the rod type (smooth and threaded), bond length, and the material used to fill the groove (type of mortars). It was found that threaded rods outperformed all other types investigated, and pull-out strengths can be significantly improved through careful selection and optimization of the mortar type and bond length.

## 1. Introduction

Masonry encompasses a diverse array of structures, such as bridges and homes, with both functional and heritage value. Nevertheless, these constructions are vulnerable to degradation from environmental factors and seismic activity, necessitating advancements in conservation and reinforcement techniques, which would ensure the continued re-use of these aging structures.

**Figure 1:**
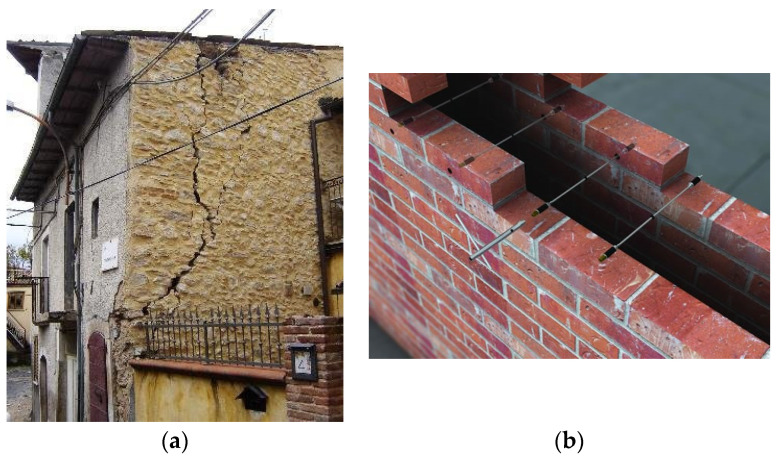
(**a**) Development of out-of-plane damage cracking in a masonry structure; (**b**) dry steel rod ties providing cross-lateral tensile reinforcement in a cavity wall.

The deterioration of masonry structures poses significant risks to safety and heritage, with climate change, extreme weathering, and seismic damage [1,2,3,4]. These structures form a substantial part of global architectural heritage, requiring effective preservation strategies. Recent earthquakes in Turkey (2023) (moment magnitude M_w_ = 7.8), Afghanistan (2022) (M_w_ = 6.3) and Croatia (2020) (M_w_ = 5.3) have demonstrated again the weaknesses of masonry against seismic forces. Out-of-plane failure mode is frequently observed in historic masonry buildings, where entire walls behave like rigid segments, independent of the primary structure (Figure 1a). Collapse is primarily induced when elements of vulnerability, such as missing, deteriorated, or improper connections between walls and between floors are present [5,6] or by the low quality of the masonry material, especially against actions inducing tensile stresses in masonry [7].

In historic buildings, seismic reinforcement aims to activate the so-called “box-like” behaviour. This includes concrete or steel ring beams and punctual connection elements tying opposing walls (commonly steel bars fixed with endplates) (Figure 1b), injected steel bar anchors placed into boreholes and filled with pressurized cement or lime mortar to connect wall leaves. While the concrete ring beam system is effective in improving local and global masonry structural behavior, it is found to be unsuitable in many instances as it often requires the roof to be dismantled.

Dry tying [8,9] is an effective method for improving seismic resistance in historic masonry buildings and heritage buildings and has been employed as a retrofitting system. End-anchored steel bars, which are prone to corrosion, leading to reduced effectiveness over time, are often used in these applications. The use of Fibre Reinforced Polymers (FRP) ties has recently gained traction [10,11]. Higher tensile properties of FRP materials highlight the benefit of less invasiveness [12], which is allowed for by the smaller dimensions of reinforcement required to produce the same effect, meaning smaller boreholes can be drilled in the existing masonry. The use of FRPs also provides the benefit of corrosion resistance, improving the system’s durability over time. Successful applications of these systems in masonry can be found in the existing literature [13,14,15,16,17,18]. Numerical modeling and the utilization of nonlinear interface elements serve as an efficient method for studying the interaction between various constitutive elements of solids. This approach effectively captures failure mechanisms across a wide range of structural systems, including both unreinforced and reinforced masonry [19,20,21].

Another interesting material is high-strength titanium alloy (TA) in the form of rods used as injected anchors in masonry structures. Titanium is rarely used within construction engineering, often being limited to medical or aerospace applications; however, its properties mean it could prove ideal for use in conservation engineering roles. Titanium is available in both commercially pure grades and titanium alloy grades but is typically used in the form of an alloy due to its superior characteristics, such as specific strength, ductility, density, and formability, which can be altered and improved by the addition of a variety of elements such as aluminum, molybdenum, vanadium, and zirconium [22].

Titanium alloys are divided into grades. In civil and other engineering applications, the most used and readily available TA grade is Ti-6Al-4V (grade 5). This grade of titanium alloy boasts a specific strength approximately four times greater than that of S275 carbon steel and presents elastic deformations and yield strengths considerably higher than S275 (Table 1). 

Considering the mechanical, thermal, and corrosive resistant properties of titanium alloys, they may prove viable in reinforcement in masonry buildings. From a mechanical standpoint, titanium alloys show great advantages over typical S275 steel in this application. The lower Young’s Modulus of titanium allows for greater ductility, potentially improving a masonry structure’s global box behavior and reducing the risk of local damage mechanisms by improving the structure’s overall ductility and energy dissipation under cyclic action, which could prove particularly beneficial in resisting seismic loading. Titanium’s high yield strength combined with low density means its specific strength is greatly superior to that of steel, proving another benefit in the area of earthquake engineering. 

Although the current use of titanium within civil engineering is small-scale, its applications and the volume of its use are growing for both concrete [25,26,27,28,29] and masonry structures [30,31,32,33,34,35,36,37]. The most significant factor contributing to this growth is the limitations of carbon steel in long-life weather-exposed structures being highlighted by recent corrosion-related collapses around the world [38,39]. The cost of titanium and its alloys has seen a remarkable decrease in recent decades. Since reaching its highest point in 2005, the cost has plummeted by around 80%. In comparison to its average cost, adjusted for inflation, during the 1990s, titanium expenses declined by 70% [33]. It is important to note that in a reinforcement project for a historic masonry structure, the material cost represents only a minor portion of the total expense, which is primarily driven by labor costs. Furthermore, the amount of titanium utilized in BJR reinforcement is relatively modest (0.5–1 kg/m^2^ of wall) [37,40,41]. Employing a costly material such as titanium might lead to a marginal rise in the overall intervention cost. The continuous emergence of infrastructure failures due to steel corrosion has led to a decrease in confidence in the material for long-lifespan structures, with titanium posing as a possible alternative. 

In the area of conservation and earthquake engineering, titanium has garnered attention for its low thermal expansion coefficient (8–9 × 10^−6^ °C^−1^). This becomes particularly interesting when considering its use in masonry reinforcement, as masonry exhibits comparable thermal expansion coefficients. The utilization of titanium and metals, in general, frequently ensures the “reversibility” of interventions, fulfilling a requirement of conservation bodies. 

There is a wealth of literature available on reinforcing stone and brickwork buildings using the BJR method. BJR involves removing the deteriorated mortar from the horizontal joints of the building’s shear walls and replacing it with new mortar. Additionally, the joints are “reinforced” with long (up to 3 m) ribbed or helical rods. Previous studies have also explored the use of stainless steel for these purposes [39,40]. 

Drobiec [42] studied reinforced masonry brickwork walls subjected to axial compression, which are presented. As reinforcement, smooth and spiral twisted steel rods were used: a limited increase in masonry compressive strength was found. Ismail et al. [43] tested in shear a total of 17 walls, each being 1.2 m × 1.2 m in size. Results were studied in terms of failure modes, shear strength, maximum drift, pseudo-ductility, and shear modulus. Walls reinforced with twisted stainless steel rods failed along distributed diagonal cracks in a more ductile fashion and exhibited a shear strength increment ranging from 114% to 189%. Castori et al. [44] employed identical titanium rods to those used in this study. They discovered that utilizing a lime repointing mortar, which is generally lacking in mechanical strength, could significantly undermine the reinforcement effectiveness of the titanium rods.

This paper evaluates the use of titanium rods in cement and lime mortars as a reinforcement for masonry. Past research in the literature does not include studies of the bond between titanium rods and mortars. This paper aims to fill this problem and address this gap in research. 

To study the bond, pull-out tests [45] were executed on threaded and unthreaded titanium rods set in cement and lime mortars inside predrilled clay bricks to various lengths. The main objective was to analyze the bond strength achieved between the titanium rods and the clay bricks with different variables, to assess the potential for the use of this reinforcement method with respect to real-world factors and to assess any potential benefits or implications this may incur for the fields of earthquake and conservation engineering.

## 2. Materials and Methods

Experimental campaign aims to study the bond between titanium rods embedded into an inorganic matrix, i.e., a cement or lime mortar. The pull-out testing is being carried out as part of a study to collect quantitative data on the bond strength between titanium and masonry, which will then be used to assess the suitability of masonry reinforcement with titanium rods.

Bricks utilized in pull-out tests were sourced from the same batch and subjected to mechanical characterization. Two kinds of mortar (lime and cement) were employed for bonding, along with smooth and threaded rods. The hole diameter in the bricks was maintained at 14 mm, while the embedment length ranged from 50 mm to 100 mm. All other testing parameters (such as test method, load rate, and rod application technique) were consistent across all samples.

### 2.1. Clay Bricks

In historical buildings, the sizes of fired bricks differed across regions and historical periods. In the UK, although the length (approximately 215 mm, 8.5 inches) and width (102.5 mm, 4 inches) have stayed relatively consistent over the past two centuries, the thickness could vary between 50 and 80 mm. For our experiment, we utilized bricks with standardized dimensions in line with industry and building norms [46]. These are the bricks commonly employed in modern brickwork constructions. 

**Figure 2:**
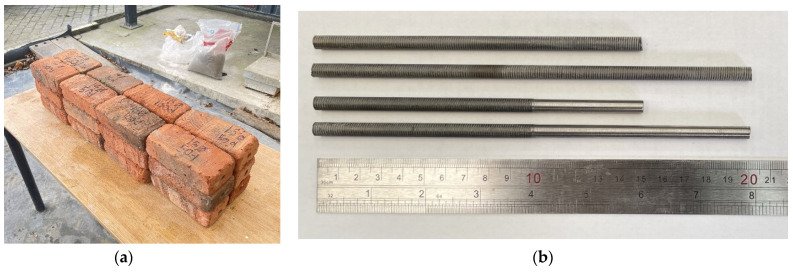
(**a**) The clay bricks used in the experiment, with their format labelling; (**b**) threaded and smooth titanium rods.

Pull-out testing exclusively employed solid bricks, as they are prevalent in historical structures frequently requiring retrofitting. The bricks used in this experiment are Class 1 [45] clay solid bricks of dimensions 215 × 102.5 × 65 mm (Figure 2a). Each brick was inspected for stress fractures and/or excessive degradation prior to testing [47]. 

Standard non-frogged (without indentation) solid bricks used in construction in the United Kingdom were employed for pull-out testing. Their main properties are presented in Table 2. The average compressive strength was 28.4 MPa, with a coefficient of variation of 15.6%. 

### 2.2. Titanium Rods

The bed joints in historical brickwork generally measure 8–12 mm in thickness. Due to this, the rods required for BJR should be smaller to fit into the bed joints alongside the repointing mortar [48,49]. The rods used in this experiment are Grade 5 titanium alloy (Ti-6Al-4V), shown in Figure 2b. The rod dimensions prior to preparation have an external thread diameter of 6.35 mm, with a length of 200 mm and mechanical properties summarized in Table 3. Titanium rods were produced and provided by Perryman company (Houston, PA, USA).

### 2.3. Lime Mortar

**Figure 3:**
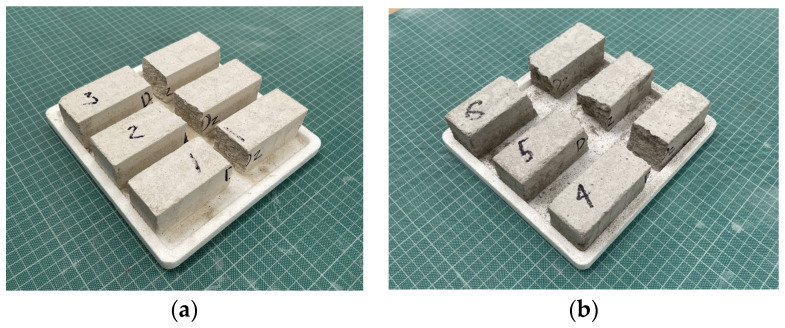
(**a**) Lime mortar prisms and (**b**) cement mortar prisms after flexural testing.

Traditional masonry in historical buildings always uses lime mortar. Therefore, the first choice was made to employ lime mortar for bonding the titanium rods to the masonry, ensuring high compatibility with the traditional masonry materials. The lime mortar was made of a mix design of 5 kg clay lime, 2 kg fine building sand, and 0.75 dm³ of water. The lime used in the mix was Class M-15 [50]. Sample prisms (160 × 40 × 40 mm) were poured to undergo material testing for the lime mortars [51], as shown in Figure 3.

Compressive and flexural testing was carried out on prism samples of the lime mortar. The mechanical properties and test results are shown in Table 4. 

### 2.4. Cement Mortar

Considering the relatively low mechanical properties of lime mortars and the significant shear stresses experienced at the mortar-to-rod interface in a pull-out test, it was determined necessary to additionally utilize a stronger cement mortar for bonding the rods to the bricks. The cement mortar used in this experiment was created with a mix design of 1 kg of Portland Cement, 4 kg of fine building sand, and 1 dm³ of water. Sample prisms were again poured for material testing of the cement mortar, shown in Figure 3b. The cement mortar underwent compressive and flexural testing to determine its mechanical properties (Table 3).

### 2.5. Test Method and Instrumentation

**Figure 4:**
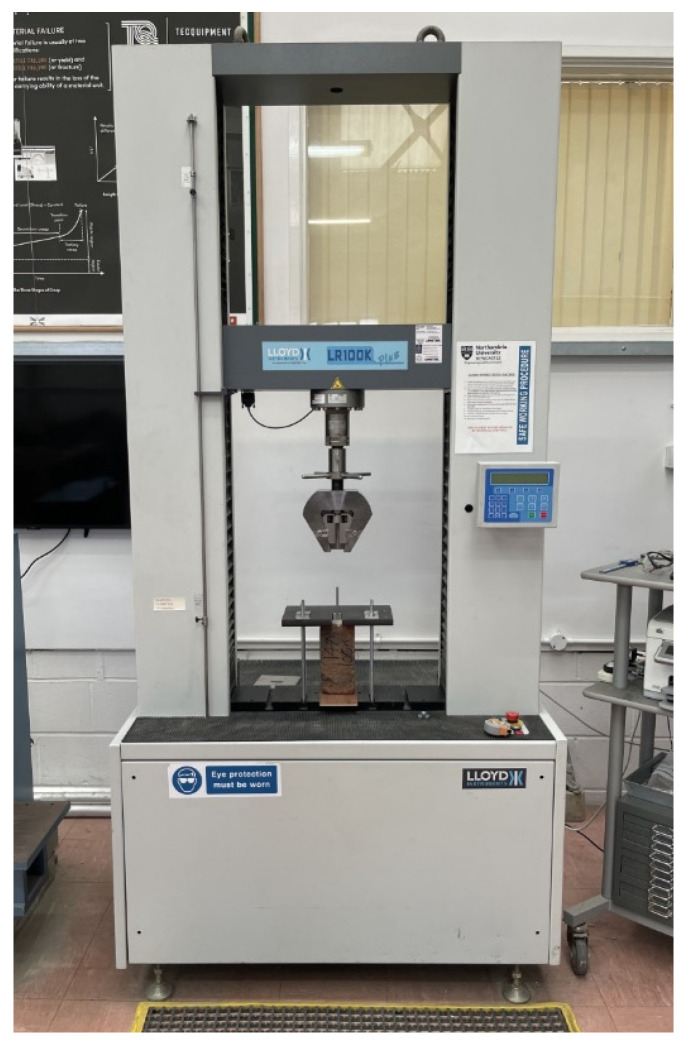
The LR100k Plus universal testing machine used to carry out the pull-out tests.

Tests were carried out at Northumbria University laboratories at a temperature between 20 and 22 °C, with humidity levels between 35% and 45%. The instrumentation used in this pull-out test is the LR100k Plus Universal Testing Machine (Ametek, Bognor Regis, UK) shown in Figure 4. For the pull-out test, the machine was set up to comply with ASTM E488 [52], the standard test method for the strength of anchors in concrete elements, which can also be applied to the masonry unit used in this test.

Grade 5 threaded titanium rods were used in this experiment. 24 rods were cut to 150 mm (12 rods) and 200 mm (12 rods) lengths. The diameter of 12 rods was reduced to 6 mm smooth. 

When machining the rods, coolant was used during both cutting and turning processes to prevent alteration of the titanium’s mechanical properties through excessive heating and superficial hardening. Once the rods were machined to size, they were individually cleaned to remove any debris between the threading. 

### 2.6. Preparation of Clay Bricks

The bricks used in this experiment are UK standard clay bricks, with dimensions of 215 × 102.5 × 65 mm. Before preparation, each brick was inspected. They were then labeled in accordance with their prospective drill depth and gauge, of which the following formats would be produced: hole diameters were 14 or 16 mm, and hole depths were 50 or 100 mm. 

**Figure 5:**
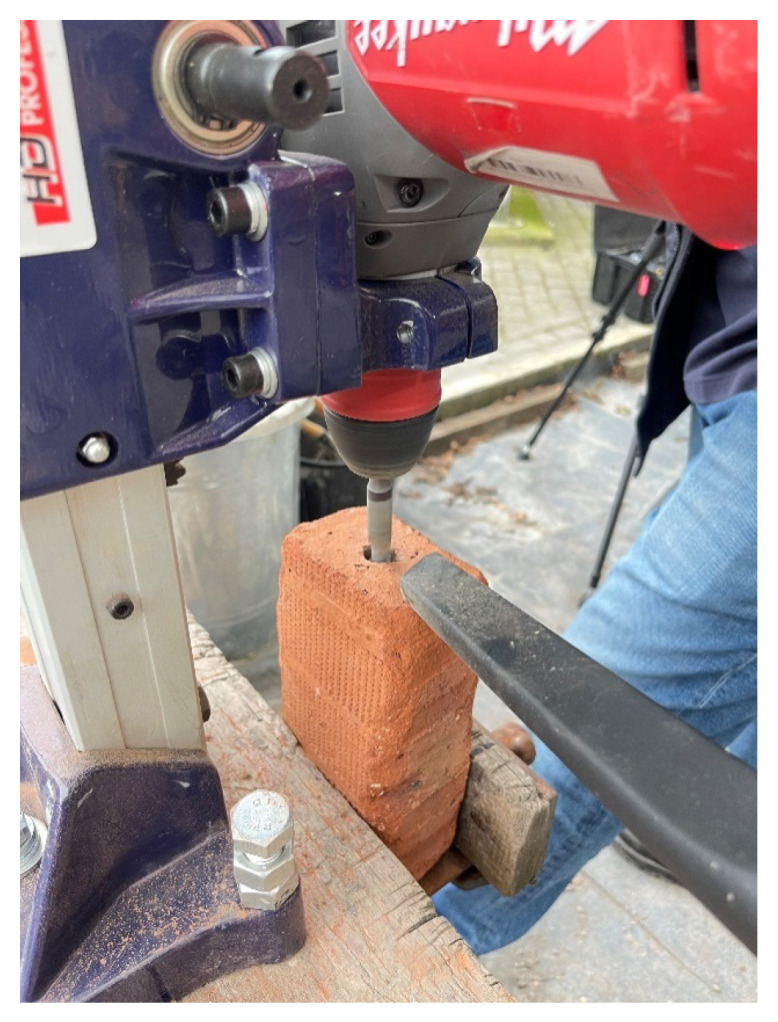
Drilling the hole in the clay bricks.

Figure 5 depicts the setup used for drilling the brick samples. On a flat workbench, a Milwaukee SDS drill was set up within a pillar stand. A 14 mm drill bit was inserted and checked for 90° by use of a spirit level. For each hole depth (50 mm, 100 mm), the drill bit was taped off to ensure the drill depth was equal on each operation. Individually, the bricks were then inserted into the clamp with the drill bit centric to the face and tightened. The brick was also checked for 90° with the spirit level. 

Once satisfied with the drill and brick positioning, each brick was individually drilled down to its specified embedment length, ensured by the drill marking. During drilling, a vacuum was positioned over the drilling site to remove dust and prevent excessive heating. After each brick was drilled, compressed air and water were used to clean out the borehole. 

### 2.7. Casting

**Figure 6:**
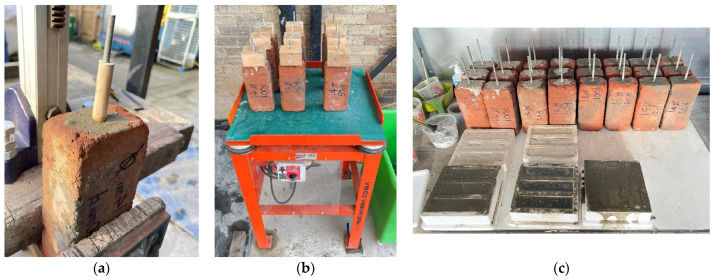
(**a**) application of the tamping rod; (**b**) samples in the vibrating table; (**c**) casted samples stored in a ventilated room.

Before mortar casting, each brick sample was soaked in water for a period of 10 min to fully saturate, preventing moisture from egressing from the mortar into the brick pores and improving overall bond strength. The specific mortar mix for each sample was poured into the borehole to fill up to halfway. The sample’s corresponding titanium rod was then inserted into the borehole before the surrounding mortar was tamped down with the use of a bored tamping rod. This process was repeated, adding mortar and tamping until the void surrounding the rod was completely filled with mortar. At the end of this process, the rod protrusion was measured to ensure the rod was positioned down to the base of the borehole. Figure 6a shows the process and the application of the tamping rod to ensure complete void infill and that all the samples are ready for testing. 

The rod was checked for vertical alignment with a spirit level before the sample was placed onto the vibrating table for a period of 5 min at 3000 vibrations per minute. Drilled wooden blocks were placed over the rod protrusion during vibration to prevent movement. After vibration, the rod was again checked for vertical alignment to ensure no translation or rotation had occurred during the vibration. This process was then repeated for all samples (Figure 6b).

Once the samples were cast, vibrated, and checked for alignment, they were placed in a locked, ventilated storage unit for the duration of their respective casting periods (Figure 6c). The cement and lime mortar samples were given a 30-day and a 60-day curing period, respectively, to ensure complete curing was achieved prior to the pull-out testing. 

### 2.8. Pull-Out Testing

**Figure 7:**
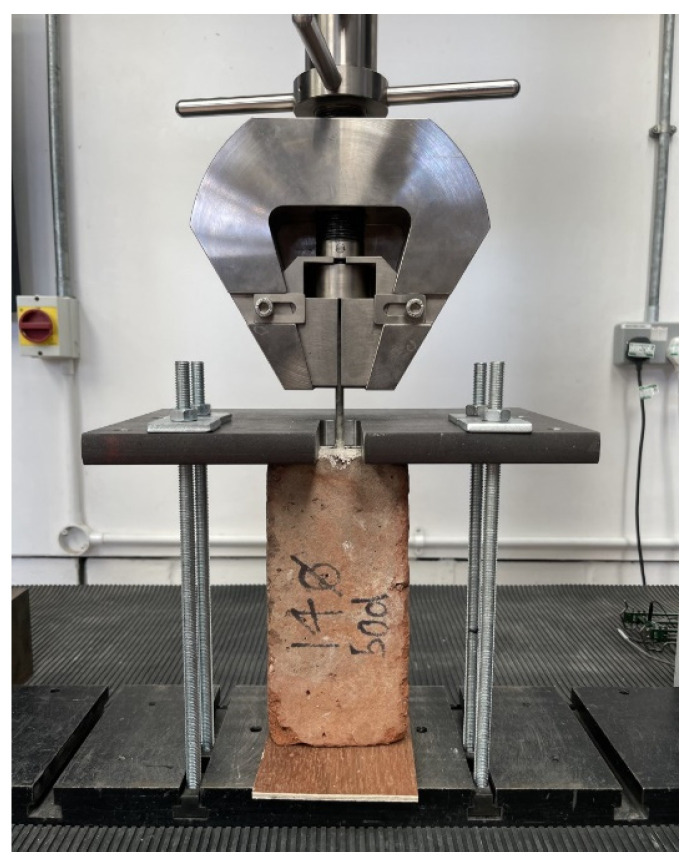
The positioning and securement of the samples prior to commencing load application.

A securing jig was assembled on the testing machine to ensure specimens were fixed and precisely aligned along the vertical axis. Subsequently, the specimens were carefully placed atop a plywood element. A steel plate was then positioned over the brick specimen. This assembly was secured using washers and nuts, with particular attention paid to ensuring that the nuts were uniformly tightened to maintain the assembly’s stability. The vertical alignment of the setup was verified post-tightening. The testing machine’s jaw was then lowered to encircle the rod, followed by a tightening process. The vertical alignment was re-examined after this adjustment to ensure the specimen’s optimal positioning. Figure 7 illustrates the arrangement and securing of the specimens within the testing apparatus prior to the application of the load.

Upon the specimen’s installation in the testing machine, an initial preload of 5 N was applied. The tensile test commenced thereafter, proceeding at a controlled rate of 1 mm/minute until the specimen’s failure or until reaching a maximum extension of 4 mm, whichever occurred first. The NexygenPlus 4.0 software [53] was employed to capture and record the experimental data and to oversee the operation of the testing machine throughout this process.

The experimental setup created at the interface between loading steel plates and specimen during pull-out additional compressive stresses in the bricks that counteract the dilation of the clay brick. Clearly, this is a limitation of the experimental setup; however, this compressive stress is very limited. The latter dilation is caused by the longitudinal translation of the rod threads, thereby generating passive confinement that artificially enhances the development capacity of the pulled bar. The maximum pullout load was about 6–8 kN, corresponding to a compressive stress of 0.9–1 MPa, less than 5% of the brick compressive strength. It is also important to note that an area (25 × 80 mm) near the holes in the bricks was unconfined (not being in contact with the steel plate, see Figure 7). Based on this, we believe that the effect of the compressive stress is very limited.

## 3. Results and Discussion

The objective of the pull-out tests was to determine the shear bond strength at two critical interfaces: between the rod and mortar, and between the mortar and brick, to identify the failure modes and pull-out capacities. The results, including the maximum load-bearing capacity prior to shear failure and the corresponding shear bond strength, provide valuable insights into the mechanical performance of the reinforcement system. These insights are instrumental in identifying areas for design or construction enhancements. Additionally, the analysis of load versus slippage curves enabled an evaluation of additional information, such as post-elastic behavior, stiffness, and failure mechanisms, thereby contributing to our understanding of its effectiveness in practical applications.

The experimental protocol involved conducting pull-out tests on 24 samples, encompassing variations in mortar types (cement and lime), rod textures (threaded and smooth), and embedment lengths (50 mm and 100 mm). Each configuration was represented by three samples to ensure the reliability of the findings. Table 5 summarizes the processed data collected from each test, providing an overview of the experiment’s outcomes. Pull-out test results account for the reinforcement system’s mechanical behavior and underscore its potential for real-world application, thereby affirming its practical viability.

### 3.1. Pull-Out and Bond Shear Strength

Table 5 summarizes the average maximum pull-out load and bond shear strength for the samples, categorized by their specific variables. The data presented in Table 5 reveal considerable variability in both maximum pull-out load and bond shear strength across the samples tested. This confirms the importance of the strength of the mortar and rod texture of the rod in the stress transfer. The range of maximum pull-out loads is notably broad, with the highest load observed for sample S10 at 7428 N (bond strength: 3.725 MPa), and the lowest for sample S3 at only 138 N (bond strength: 0.146 MPa). 

**Figure 8:**
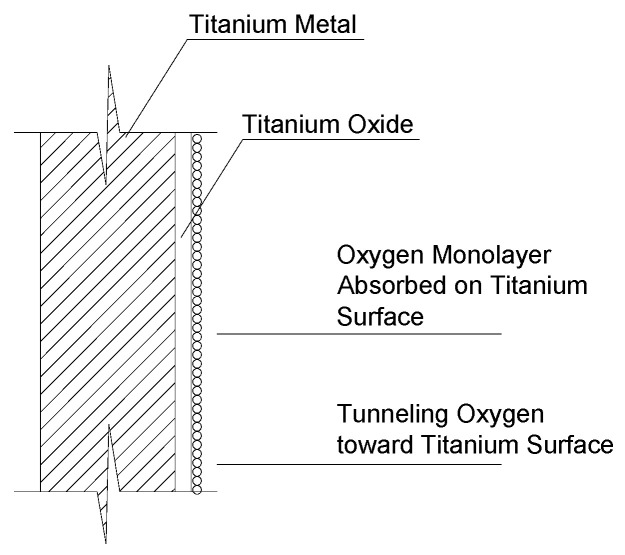
The durability of titanium is facilitated by oxide film formation.

An interesting result of this experimental work regards the effect of rod`s thread on the pull-out capacity. It was expected that threaded rods outperform smooth rods in both maximum pull-out load and bond shear strength, but the very low pull-out loads of smooth rods are relevant and remarkable. It is worth recalling that titanium resistance to corrosion is due to the formation of a passivation film when exposed to atmosphere. The passivation film (Figure 8) is very stable and has excellent corrosion resistance. However, this film may highly reduce the bond with mortar and facilitate rod slippage in BJR interventions, compromising the reinforcement effect. The yielding tensile load of the rods used in this experiment is 26.1 kN (for a 6 mm diameter rod), but the pull-out load is only 0.243 kN (for 50 mm length) and 0.398 kN (for 100 mm embedment length in a cement mortar). This is clearly an indication of an incorrect application, where a strong reinforcement material (titanium) is unable to contribute to reinforcing the masonry due to the low bond strength. The first conclusion of this experimental work is that smooth rods should not be used in BJR applications as the tensile resistance of the titanium rods cannot be activated due to a very low mortar-to-rod bond strength.

Analysis of the data reveals that threaded rods achieved high pull-out capacities, resulting in an average shear bond strength of 1.961 MPa across all threaded samples, as opposed to 0.707 MPa for smooth samples. This distinction underscores the effectiveness of threaded rods in reinforcing masonry structures, highlighting their potential for improving structural integrity.

Analysis of the results also indicates a superiority in the performance of cement mortar samples over those composed of lime mortar. The observed differences are important, suggesting that the performance enhancement provided by the cement mortar, together with the use of threaded rods, in this context is significant. For example, for a 50 mm embedment length, the pull-out capacity was 2630 N (2.673 MPa bond strength) for threaded rods in cement mortar, compared to 1358 N (1.362 MPa bond strength) when threaded rods in lime mortar were used. 

The data in Table 5 and Table 6 also underscore a significant scattering in the bond strength of smooth rods. Coefficients of variation (CoV) are given in brackets in Table 6. A comprehensive review of the dataset for smooth rods suggests that such variability is intrinsic: a plausible explanation for this inconsistency lies in the inherent morphologic differences between smooth and threaded rods. Threaded rods engage more effectively with the mortar due to their threads (mechanical interlocking), which naturally key into the surrounding material. In contrast, smooth rods lack this mechanical interlocking, making their performance potentially susceptible to minor variations in rod geometry, surface irregularities, contamination, voids in mortar, or differential smoothness, leading to unpredictably varied results. For smooth rods, rod-to-mortar bonding is mainly guaranteed only by chemical links between the materials (titanium and mortar). This produced a high scattering in the test results, with CoV values up to 85%. 

It is worth noting that the thread depth of the titanium rods was small (0.175 mm). It is posited that increasing the thread depth could further enhance the bond shear strength, suggesting a direct correlation between thread depth and bond performance. This outcome was anticipated, with threaded rods significantly surpassing smooth rods in terms of maximum pull-out capacity and bond shear strength. Future research could beneficially explore the effects of varying thread depths or other superficial rod treatments (sandblasting, ribs, curling on performance), potentially offering insights into optimizing rod design for practical BJR applications.

Furthermore, the influence of embedment length L_b_ on reinforcement performance was also relevant; a significant increase in pull-out capacity was recorded as the embedment length L_b_ increased, due to the enlargement of the available bond area. This behavior seems governed by a limit bond strength value of about 2–2.5 and 3–4 MPa for lime and cement mortar, respectively. Results show that, doubling the embedment length from 50 mm to 100 mm, cement samples with threaded rods exhibited a 139% increase in maximum pull-out capacity, with the shear strength only increasing 19.2%. 

Table 6 presents the aggregated performance metrics for each sample format. This table facilitates an understanding of how various factors influence the reinforcement’s effectiveness, guiding the optimization of design for enhanced structural resilience. The data analysis shows distinct patterns and correlations within the performance metrics of each sample format, particularly highlighting the interaction between mortar type (lime or cement), embedment length and rod texture (threaded or smooth). 

Among the various configurations tested, the formats incorporating 100 mm embedment length, threaded rods, and cement mortar emerged as the most effective in terms of maximum pull-out load capacity and bond shear strength. Sample Format 4 (Cement/Threaded/100 mm) shows the highest bond shear strength at 3.213 MPa and pull-out capacity (6410 N). Test results suggest that the mechanical interlock facilitated by the rod’s thread and the mortar’s mechanical properties are critical in optimizing bond strength. 

It is worth noting that the mechanical properties of the used mortars were very different: Table 3 shows that the compressive strength of the cement mortar (2.472 MPa) was more than double the strength of the lime mortar (1.182 MPa). The bending strength of cement mortar was 5 times bigger (1.457 MPa) compared to the lime mortar (0.287 MPa). This had a significant effect on the mortar-to-rod bonding characteristics. By comparing Sample Format 4 (Cement/Threaded/100 mm) with Sample Format 8 (Lime/Threaded/100 mm), it is possible to note that the use of cement mortar caused an increase of about 55% in pull-out capacity compared to samples made with lime mortar.

### 3.2. Load vs. Displacement

The analysis of the load versus displacement curves provides an understanding of the structural behavior of the bonding. Initially, a general overview of the load-displacement behavior across all samples offers insights into system stiffness, ductility, and failure modes, followed by a more detailed examination of specific sample formats.

**Figure 9:**
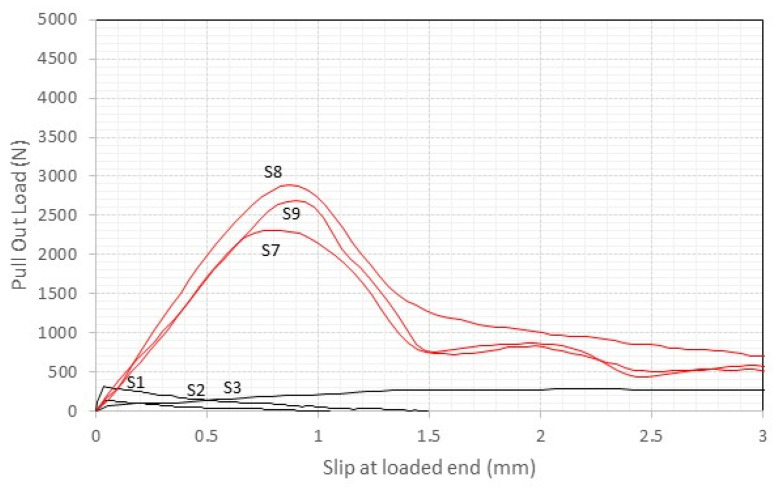
The load vs. displacement response of S1 to S3 and S7 to S9 (cement mortar, 50 mm embedment length, red lines = thread rods, black lines = smooth rods).

**Figure 10:**
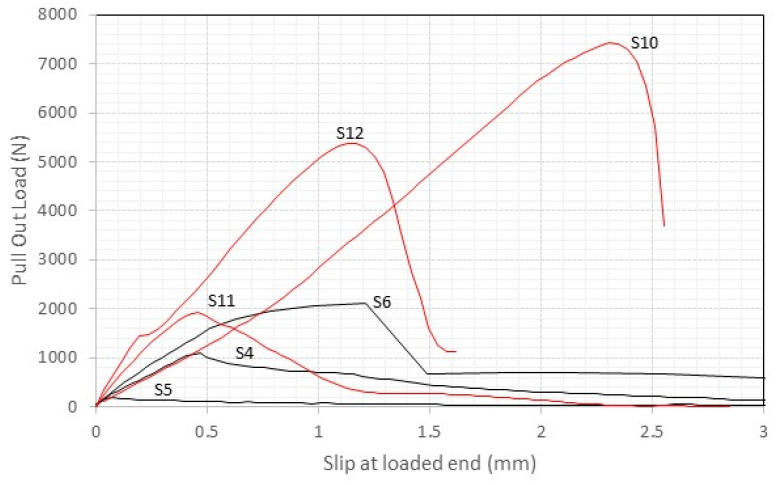
The load vs. displacement response of S4 to S6 and S10 to S12 (cement mortar, 100 mm embedment length, red lines = thread rods, black lines = smooth rods).

The force–slippage curves shown in Figure 9 and Figure 10 for the cement mortar samples (S1 to S12) reveal a notable diversity in structural response among the specimens. A prevalent observation is the consistency in failure patterns across these samples. The failure pattern indicates that the systems likely underwent very limited nonelastic deformation prior to reaching peak stress, subsequently failing in a relatively abrupt manner at the interface between the titanium rods and the surrounding mortar. The threaded rods remained bonded to the bricks up to higher pull-out loads, with an elastic phase of the pull-out curves more pronounced and evident. Insight regarding the elastic stiffness is provided by the slope of the load-slippage curves in the initial phase of the loading. This “stiffness” is governed by the Young`s moduli of both titanium rods (111.7 GPa) and used mortars (5–25 GPa). By comparing the curves shown in Figure 9 (samples with cement mortar) with the ones in Figure 10 (lime mortar), it can be noted that slippage in the elastic phase (before reaching the maximum pull-out load) was about 30% higher when lime mortars were used. The stiffness of these samples was significantly higher.

After the maximum load was reached, a significant abrupt reduction in pull-out capacity was noted. Slippage phenomena at the interface between the rods and the mortar were noted, and a residual pull-out resistance of 15–20% of the maximum load was recorded (Figure 9 and Figure 10). Tests were stopped when a machine extension of 4 mm was reached.

**Figure 11:**
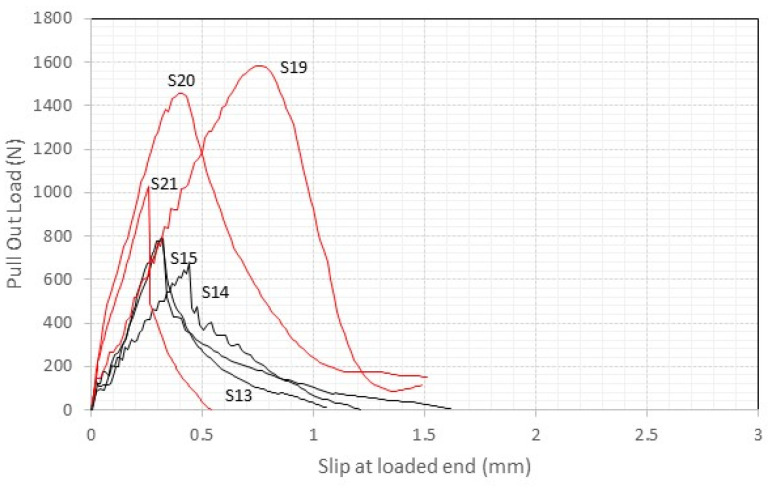
The load vs. displacement response of S13 to S15 and S19 to S21 (lime mortar, 50 mm embedment length, red lines = thread rods, black lines = smooth rods).

**Figure 12:**
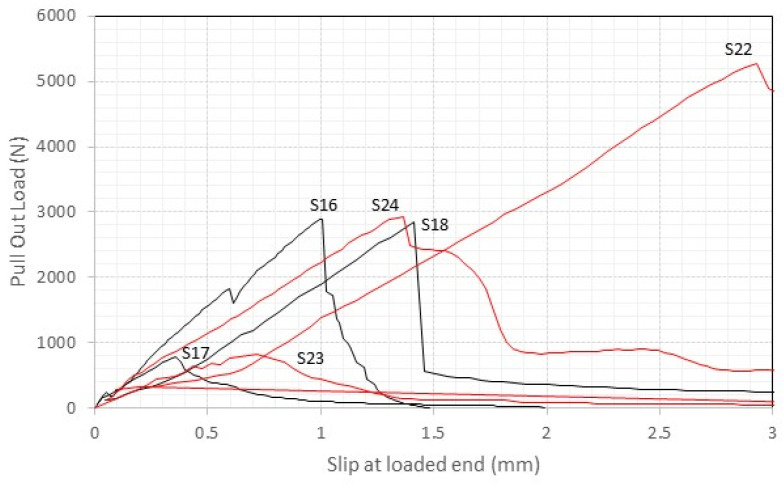
The load vs. displacement response of S16 to S18 and S22 to S24 (lime mortar, 100 mm embedment length, red lines = thread rods, black lines = smooth rods).

Conversely, Figure 11 and Figure 12, which present the load–slip data for the lime mortar samples, highlight distinct structural response. In contrast to the cement samples, the lime mortar specimens predominantly exhibit high levels of slippage at maximum pull-out load, and a more brittle failure mode, characterized by a sharp decline in force immediately following peak pull-out load. Moreover, the initial slopes of the lime sample graphs are shallower than those observed for cement, indicating reduced system stiffness. 

This comparative analysis highlights the importance of the mortar mechanical properties influencing the reinforcement system’s mechanical performance and the cement mortar ability to stiffen the bond. On the other hand, the lime mortar samples’ clearer tendency towards brittle failure and reduced stiffness highlights the critical role of mortar selection in influencing the overall behavior of masonry reinforcement systems.

### 3.3. Failure Mode

A notable observation across all samples is that bond failure consistently occurred at the rod–mortar interface, highlighting a critical area for future research and optimization. This failure mode was partially expected: if we consider, on the horizontal cross-section of the brick samples, the linear development of adhesion at the titanium rod–mortar interface (about 20 mm) and that at the brick–mortar interface (44 mm), it can be noted that the tangential stresses, for the same level of pull-out load, at the interface with the brick are over 55% lower.

**Figure 13:**
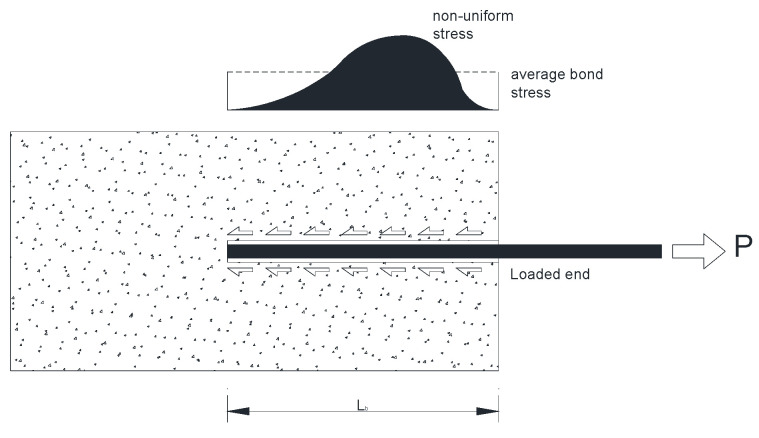
The nonuniform distribution of the bond stresses along the bonded length.

The results of the pull-out tests (Table 5) reveal critical insights into the failure mechanisms. A consistent pattern observed across all samples, irrespective of the mortar type used (cement or lime), is the localization of bond failure at the rod–mortar interface (Figure 13). No failure was noted at the mortar-to-brick interface. This phenomenon indicates that the bond strength at this interface is also weaker than at the mortar-brick interface. Such a finding underscores a pivotal area for potential enhancements in reinforcement design, specifically targeting the rod–mortar bond strength to mitigate the primary failure mode.

By comparing the tensile capacity of the rods (26.1 kN) with the pull-out capacity of the threaded rods with a 100 mm embedment length (4–5 kN), it is evident that titanium rods are insufficiently exploited. The maximum exploitation ratio (pull-out load/rod tensile capacity) is 24.6% for threaded rods, and 7.7% for smooth ones.

By comparing the test results for 50 mm and 100 mm of embedment length L_b_, it can be observed that the pull-out capacity roughly doubled when the embedment length was doubled, with a linear relationship. However, more tests are needed to verify if a longer embedment length can significantly increase the pull-out capacity. Existing literature data regarding embedment lengths in pull-out tests indicate that there is a “critical value” of embedment length: this is the critical length beyond which the developed force can no longer increase [54]. It is typically expressed as a multiple of the rod diameter and it is highly affected by the strength grade of the mortar. Critical length values in terms L_b_/d ratio are in the range 15–25, where d is the rod diameter. The average bond strength decreases when embedment lengths bigger than the critical bonded length value are used due to the nonuniform distribution of the bond stresses along the bonded length [55].

To enhance the exploitation ratio, either the pull-out capacity should be increased or smaller rods (with reduced tensile load capacity) should be employed. Therefore, it is important to study and test different rod textures, for example with ribs or other superficial deformations able to increase further the bonding to mortar. The use of titanium threaded tubes, made with a smaller amount of titanium material and characterized by a smaller tensile capacity compared to solid rods, could also be considered, and tested, but it has to be mentioned that the outer diameter of the tubes cannot be larger than 6–7 mm as it can be impossible to install the rods in the bed joints of the most types of brickwork walls.

**Figure 14:**
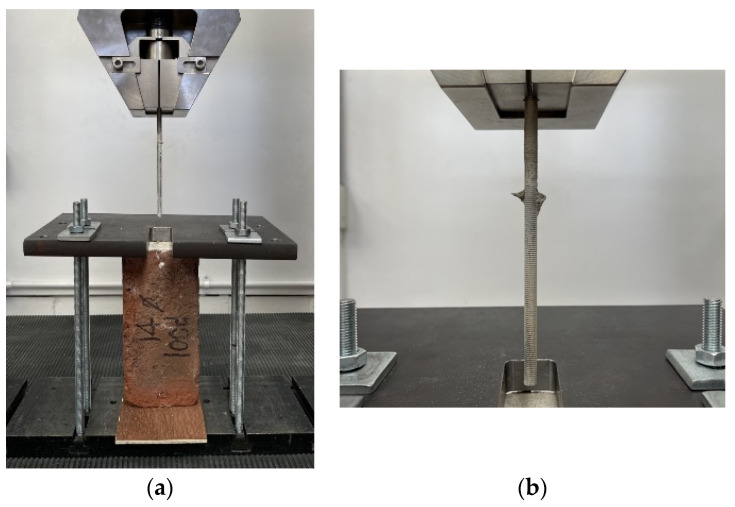
Failure at the rod–mortar interface: (**a**) test layout after complete pull-off of titanium rod, (**b**) detail of the rod.

The occurrence of adhesive failure exclusively at the rod–mortar interface, characterized by a rough, irregular fracture surface (with remains of mortar adhering to the rod post-failure) further validates the comparative strength superiority of the mortar-brick bond (Figure 14). This distinction not only reinforces the need for focused improvements at the rod–mortar interface but also provides a clear direction for subsequent design optimizations.

**Figure 15:**
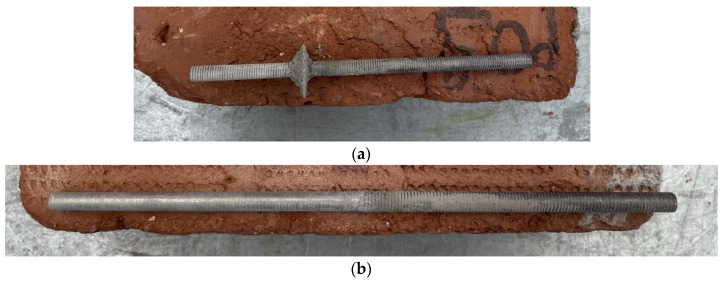
(**a**) Cone-shaped failure recorded in threaded rods, (**b**) During the tests on smooth rods, the failure occurred due to slippage, without the remaining mortar sticking to the rods.

Additionally, the presence of small, cone-shaped failure patterns at the entry point (loaded end) of the rods suggests an unconfined mechanical interlocking between the titanium rod and the surrounding mortar (Figure 15a) in this part of the bond. Figure 15b also shows the typical failure mode of smooth rods: it can be observed how the smooth bars, after the pull-out test, remain almost perfectly clean without any mortar residues. This is an indication of the weakness of the bond between the bar and the mortar, which resulted in low pull-out loads in the experimental tests.

The absence of visible deformation in the titanium rods across all tests is also particularly noteworthy, suggesting that the rods’ ultimate tensile strength far exceeds the transferable bond shear strength at the rod–mortar interface. This observation opens further research directions, potentially exploring the use of ribbed rods to increase the bond area and to add a higher mechanical interlocking between the mortar and the rods. Such experimental adaptations could significantly contribute to developing more resilient and efficient reinforcement solutions tailored to meet the structural demands of old masonry constructions.

## 4. Conclusions

BJR is a retrofitting technique where metal bars or wires are embedded between mortar joints in masonry walls. The reinforcement system is based on transferring stresses from the masonry to the reinforcing bars using a repointing mortar. This reinforcement adds tensile strength to the structure, helping distribute loads and resist cracking, improving the overall stability and durability of the wall. 

This paper investigated the viability of titanium rods and cement and lime mortars as a BJR method by conducting pull-out tests on threaded and unthreaded Grade 5 titanium rods. After analyzing the results, the following conclusions can be drawn:

- In all pull-out tests, bond failure developed. The bond failure occurred exclusively at the rod–mortar interface. No other alternative failure modes were observed in the tests, namely either pullout at the interface between brick and mortar or cracking of the mortar or brick materials.
- The structural response of smooth rods was particularly unsatisfactory, with very low pull-out loads and low exploitation ratios. Also, test results of smooth rods were extremely scattered in terms of pull-out loads due to the chemical nature of the bonding.
- The passivation film on the titanium rod surface, which guarantees excellent corrosion resistance, likely reduced the bond with mortar and facilitated rod slippage. This compromises the reinforcement effect. It can be concluded that smooth rods should not be used in BJR applications as the tensile resistance of the titanium rods cannot be activated due to very low mortar-to-rod bond strength.
- The threaded rods significantly outperformed smooth rods in both maximum pull-out load and bond strength. The use of a threaded rod in a cement mortar, with the same bond length, produced an increase in pull-out load and bond strength varying between 901 and 1510% compared to smooth rods. For rods embedded in a lime mortar the increase in pull-out capacity was smaller (77–88%) compared to smooth rods.
- Using cement mortar to bond the rods to masonry seems to be also critical. The cement mortar samples exhibited better performance with an average increase of 93.6% and 55% in maximum pull-out load for a bond length of 50 and 100 mm, respectively, compared to lime mortar samples.

In conclusion, it can be stated that the use of threaded rods with a high-strength cement-based mortar is of fundamental importance in order to effectively transfer stresses from the masonry material to the reinforcing titanium rod and activate the reinforcing action of the bars. The maximum shear stresses that have been recorded range between 2.6–3.2 MPa, corresponding to a maximum pull-out load of approximately 6.4 kN.

While more tests will be necessary to study and evaluate the critical length, beyond which the developed pull-out force can no longer increase as a consequence of the nonuniform distribution of the bond stresses along the bonded length, it can be concluded that mechanical interlock facilitated by the rod’s texture and the mortar’s inherent properties plays a critical role in optimizing bond strength. Future research could explore these dynamics further, potentially leading to refined recommendations for materials and design strategies in masonry reinforcement applications.

The practical implications of this study are noteworthy: forthcoming guidelines and building codes are advised to potentially prohibit the utilization of smooth rods in BJR applications, with careful consideration given to the selection of repointing mortar. While employing lime is undeniably aligned with the preservation principles and compatibility of both new and old materials, it is equally crucial to utilize mortar of sufficient strength to ensure, through shear, the effective transfer of stresses between the masonry and the reinforcing rods.

Future experiments could extend the investigation to include rods penetrating through multiple bricks, simulating real-world masonry wall conditions. Such studies would offer valuable insights into the BJR reinforcement’s efficacy across a wall.

## Figures and Tables

**Table 1:** Properties of the main titanium alloys in comparison to S275 steel [23,24].

Grade	UNS Designation	Young’s Modulus (GPa)	Yield Strength (MPa)	Tensile Strength (MPa)	Weight Density (kN/m^3^)	Thermal Expansion (10^−6^ °C^−1^)
Ti Grade 1	R50250	105–120	135–220	240–320	45	8.6
Ti Grade 2	R50400	105–120	275–290	345–400	45	8.6
Ti Grade 3	R50550	105–120	380–450	450–530	45	8.6
Ti Grade 4	R50700	105–120	480–580	550–680	45	8.6
Ti Grade 5 Ti-6Al-4V	R56400	95–115	800–900	900–1050	44	9.0
Steel (S275)		206–220	275–320	370–450	78.5	12.0

**Table 2:** Characteristics and mechanical properties of solid bricks used for pull-out tests. Coefficient of variation in ( ).

Weight Density (kg/m^3^)	1723.8 (3.9)
Material	Clay
Type	Solid
Dimensions (mm)	215 × 102.5 × 65
Failure Compression Load (kN)	636
Compressive Strength (MPa)	28.4 (15.6)

**Table 3:** Mechanical characteristics of the titanium rods. Coefficient of Variation in ( ).

Titanium Grade	5 (Ti-6Al-4V)
Rod diameter (mm)	6.35 *–6.0 **
Linear Density (kg/m)	0.144
Young’s Modulus (GPa)	111.7 (5.1)
Yield Strength (MPa)	923 (7.3)
Tensile Strength (MPa)	1012.6 (9.1)

* threaded rods, ** smooth rods.

**Table 4:** Flexural and compressive properties of mortars. Coefficients of variation in ( ).

Sample ID	Mortar Type	Compressive Strength (MPa)	Bending Strength (MPa)	Young’s Modulus in Bending (MPa)
M1	Lime	1.171	0.32	123.11
M2	Lime	1.329	0.24	187.86
M3	Lime	1.046	0.30	167.22
		1.182 (12.0)	0.287 (14.5)	159.4 (20.8)
C4	Cement	2.504	1.47	4764
C5	Cement	2.440	1.44	6730
C6	Cement	2.471	1.46	3789
		2.472 (1.3)	1.457 (1.0)	5094 (29.4)

**Table 5:** Summary of the pull-out tests results.

Sample ID	Mortar Type	Rod Type	Embedment Length L_b_ (mm)	Max Pullout Load P (N)	Bond Shear Strength (MPa)	Failure Interface
S1	Cement	Smooth	50	309	0.328	Rod-Mortar
S2	Cement	Smooth	50	283	0.300	Rod-Mortar
S3	Cement	Smooth	50	138	0.146	Rod-Mortar
S4	Cement	Smooth	100	1103	0.585	Rod-Mortar
S5	Cement	Smooth	100	185	0.098	Rod-Mortar
S6	Cement	Smooth	100	2105	1.117	Rod-Mortar
S7	Cement	Threaded	50	2316	2.323	Rod-Mortar
S8	Cement	Threaded	50	2891	2.900	Rod-Mortar
S9	Cement	Threaded	50	2684	2.692	Rod-Mortar
S10	Cement	Threaded	100	7428	3.725	Rod-Mortar
S11	Cement	Threaded	100	1915	0.960	Rod-Mortar
S12	Cement	Threaded	100	5392	2.704	Rod-Mortar
S13	Lime	Smooth	50	801	0.850	Rod-Mortar
S14	Lime	Smooth	50	686	0.728	Rod-Mortar
S15	Lime	Smooth	50	800	0.849	Rod-Mortar
S16	Lime	Smooth	100	2900	1.539	Rod-Mortar
S17	Lime	Smooth	100	790	0.419	Rod-Mortar
S18	Lime	Smooth	100	2876	1.527	Rod-Mortar
S19	Lime	Threaded	50	1585	1.590	Rod-Mortar
S20	Lime	Threaded	50	1461	1.465	Rod-Mortar
S21	Lime	Threaded	50	1030	1.033	Rod-Mortar
S22	Lime	Threaded	100	5301	2.659	Rod-Mortar
S23	Lime	Threaded	100	814	0.408	Rod-Mortar
S24	Lime	Threaded	100	2938	1.473	Rod-Mortar

**Table 6:** Summary of maximum pull-out load and bond shear strength for each configuration.

Sample Format Type of Mortar/Rod Surface/Bond Length	Maximum Pull-Out Load P (N)	Bond Strength (MPa)	Exploitation Ratio (%)
1—Cement/Smooth/50 mm	243	0.258 (37.9)	0.93
2—Cement/Smooth/100 mm	398	0.211 (85)	4.34
3—Cement/Threaded/50 mm	2630	2.637 (11.1)	10.1
4—Cement/Threaded/100 mm	6410 *	3.213 * (22.5)	24.6
5—Lime/Smooth/50 mm	763	0.810 (8.6)	2.7
6—Lime/Smooth/100 mm	2189	1.162 (55.3)	7.7
7—Lime/Threaded/50 mm	1358	1.362 (21.4)	4.8
8—Lime/Threaded/100 mm	4120 *	2.066 * (40.6)	15.8

* one test result removed from calculation; in ( ) Coefficients of Variation (%).

## Data Availability

Data are contained within the article.
